# Supplementary material for: RAD51 Is Implicated in DNA Damage, Chemoresistance and Immune Dysregulation in Solid Tumors
Source: Cancers (Basel). 2022 Nov 20;14(22):5697. doi: 10.3390/cancers14225697 (PMC9688595; doi:10.3390/cancers14225697)
Supplement: Supplementary file 1 [file cancers-14-05697-s001.zip › cancers-1903892-supplementary.pdf]

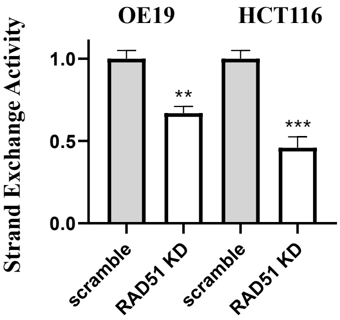

Figure S1. RAD51-knockdown inhibits homologous recombination activity. RAD51 was suppressed in OE19 and HCT116 cells using shRNAs, knockdown confirmed (shown in Figure 2B) and impact on homologous strand exchange activity evaluated using a fluorescence-based assay(** p<0.01,*** p<0.001).

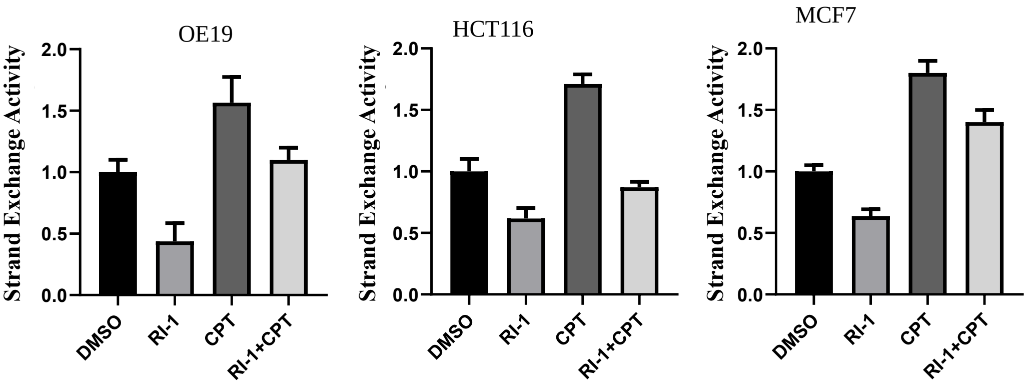

Figure S2. RAD51 inhibitor reduces camptothecin-induced homologous recombination activity. Cancer cell lines - esophageal adenocarcinoma (OE19), colon (HCT116) and breast cancer (MCF7) were treated with RAD51 inhibitor (RI-1; 20 µM), camptothecin (CPT; 100 nM) or combination of both (RI-1+CPT) for 48 hr and evaluated for impact on homologous strand exchange activity evalu

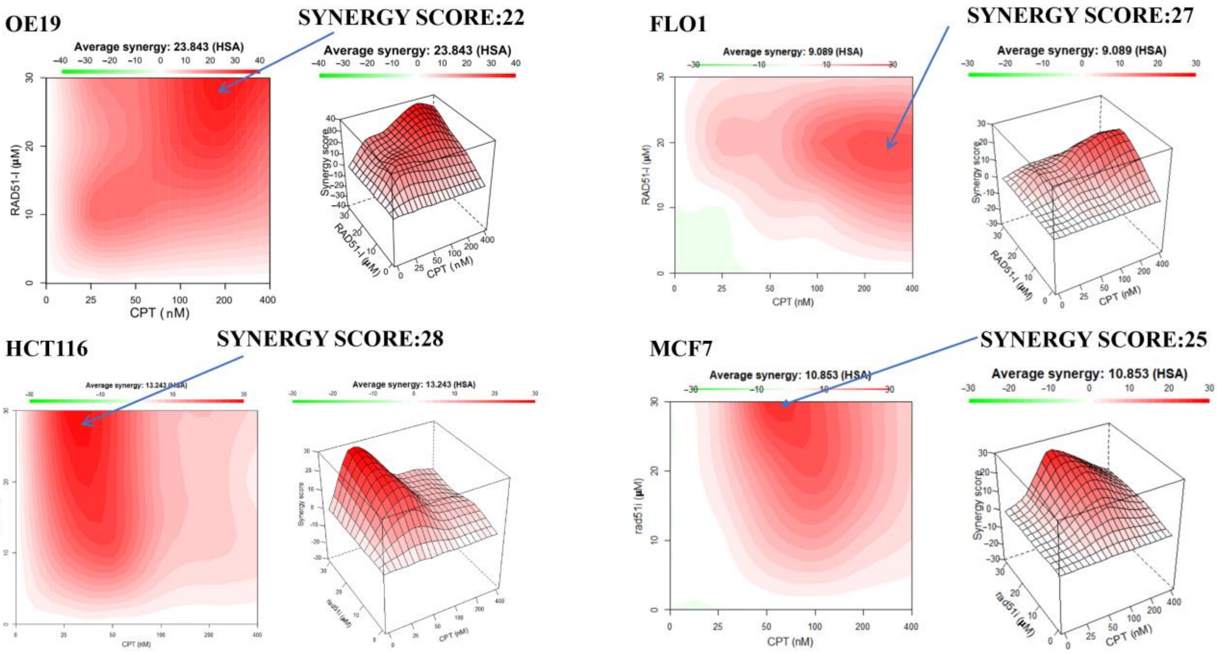

Figure S3. RAD51 inhibitor synergistically increases cytotoxicity of camptothecin in cancer cell lines. Esophageal adenocarcinoma (OE19, FLO-1), colon (HCT116) and breast (MCF7) cancer cell lines were treated with RAD51 inhibitor (RI-1; 0,10,20,30 μM), camptothecin (CPT; 0,25,50,100,200,400nM) or combination of both (RI-1+CPT) for 48 hr and cell viability assessed as shown in Figure 5. Combination index plots visualized in R environment using the HAS method in the synergy-finder package are shown. A score more than 0 (red) indicates a synergistic effect of the combination. The blue arrows in the figure indicate the region where the maximum score of the synergistic effect occurs.

A

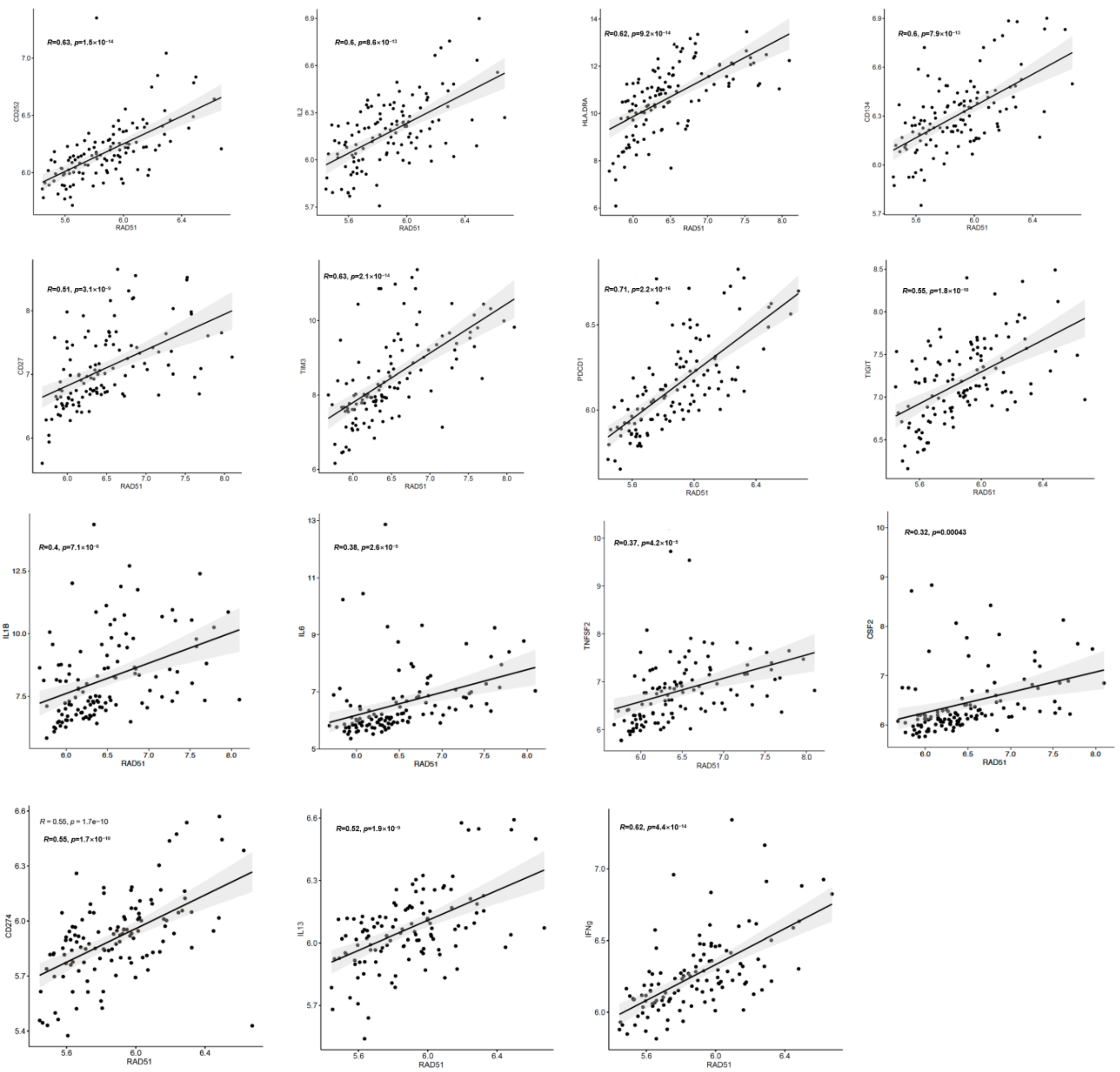

B

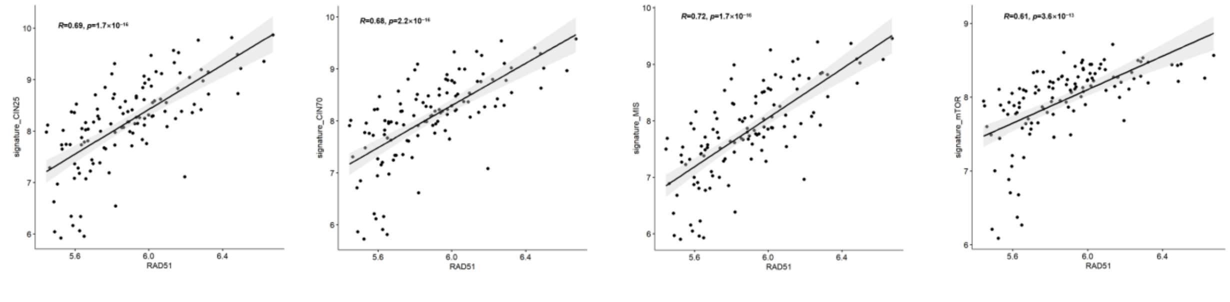

Figure S4. Correlation of RAD51 expression with immune dysregulation. RAD51 expression was evaluated for correlation with various markers of immune function in esophageal. adenocarcinoma (dataset; GSE13898). Line plots of correlation are shown.(A) Correlation of immune-related makers with RAD51 expression. (B) Correlation between immune-related pathways or signatures and RAD51 expression.

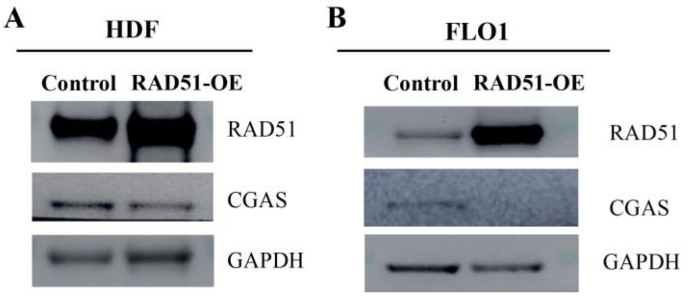

Figure S5. (A-B) Impact of RAD51 overexpression on cGAS expression. RAD51 was overexpressed in normal human diploid fibroblasts (HDF) (**A**) or esophageal adenocarcinoma (FLO-1) cells (**B**) and following selection evaluated for the impact on expression of RAD51 and cGAS using Western blotting; RAD51-OE, RAD51 overexpression.

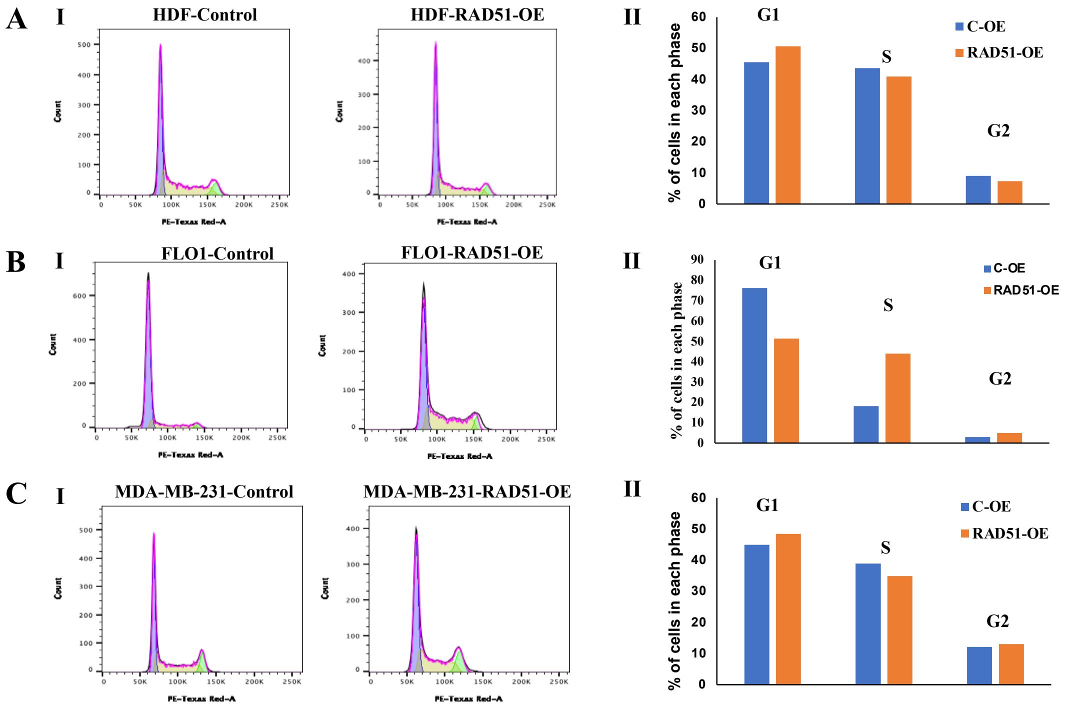

Figure S6. Impact of RAD51-overexpression on cell cycle**.** RAD51 was overexpressed in normal human diploid fibroblast (HDF) cells **(A)**, esophageal adenocarcinoma (FLO-1) **(B)** and breast cancer (MDA-MB-231) **(C)** cells and impact on cell cycle investigated using flow cytometry. Cell cycle images (panel I) and bar graphs showing percentage of cells in different phases of cell cycle (panel II) are shown.
